# Impact of comorbidity scores and lifestyle factors in curative radiotherapy in laryngeal cancer

**DOI:** 10.1007/s00066-023-02072-y

**Published:** 2023-04-12

**Authors:** Zdenka Pechacova, Radka Lohynska, Miloslav Pala, Tereza Drbohlavova, Tomas Korinek

**Affiliations:** 1 Institute of Radiation Oncology, First Faculty of Medicine, Charles University and Bulovka University Hospital, Budinova 67/2, 18081 Prague, Czech Republic; 2 Department of Oncology, First Faculty of Medicine, Charles University and Thomayer University Hospital, Prague, Czech Republic; 3 National Radiation Protection Institute, Prague, Czech Republic; 4 Military University Hospital, Prague, Czech Republic

**Keywords:** Laryngeal cancer, Radiotherapy, Concurrent chemoradiotherapy, Comorbidity, Treatment effectiveness, Organ preservation

## Abstract

**Purpose:**

The principal goal of treatment of laryngeal cancer is to eliminate a tumour while preserving laryngeal function with radio(chemo)therapy being the mainstay of treatment. The aim of this report is to present the influence of comorbidities and lifestyle factors on treatment outcomes in our cohort of patients.

**Methods:**

During the period 2009–2018, curative radio(chemo)therapy for laryngeal cancer was performed on 189 patients.

**Results:**

The median OS was 50.8 months, with a mean PFS of 96.5 months, mean LC of 101.4 months and a median follow-up of 38.1 months. Acute and late treatment toxicity grade 3–4 was observed in 39.2% patients and 10.1% patients, respectively. A significant effect on overall survival was confirmed for the baseline PS (performance status), severity of weight loss, baseline haemoglobin values, history of alcohol abuse, marital status and comorbidities according to the Charlson Comorbidity Index, as well as the ACE-27 and ASA scores.

**Conclusions:**

In our cohort of patients treated with radio(chemo)therapy for laryngeal cancer, we found good therapeutic results and an acceptable side-effect profile. Statistically significant predictors of overall survival were the baseline PS, weight loss, anaemia, associated comorbidities, history of alcohol abuse and marital status.

## Introduction

Radiation therapy is the mainstay of treatment for head and neck cancers, and, thus, for a subgroup of laryngeal cancers. In the laryngeal region, carcinomas develop in three basic localisations: supraglottis, glottis and subglottis. These areas are distinct in terms of lymphatic drainage and tumour behaviour, and, therefore, methods of treatment also differ. Laryngeal cancer is one of the less common cancers with approximately 13,430 cases (ca 10,550 men and 2880 women) per year being diagnosed in the USA [1, 2] with approximately 3620 patients dying from this disease annually. Laryngeal cancer occurs more commonly in men than in women (5.8 cases per 100,000 individuals vs 1.2 per 100,000, respectively) [1, 2]. The incidence rate in the Czech Republic is approximately 5/100,000 inhabitants and has not changed significantly in recent years [3]. The tumours occur most frequently in the 55–75 age range and affect men up to nine times more often than women [4, 5]. The main risk factors for laryngeal cancer are smoking, alcohol abuse or a frequent combination of both [2, 4, 5]. In terms of histological type, squamous cell carcinoma is usually (more than 95%) confirmed in the larynx [2, 4, 5].

When choosing a treatment method, the principal therapeutic goal is to eliminate the tumour while preserving laryngeal function with a range of parameters evaluated: the extent of the disease, the exact location, the nature of the growth, the patient’s performance status, patient preference and the ability to cooperate with treatment and the post-treatment follow-up. The treatment approach is designed by a multidisciplinary team of experienced specialists and considers the patient’s preferences. A number of professional guidelines are available: the American Society of Clinical Oncology (ASCO) summary recommendations, updated in 2018, which focus on the criteria for the choice of larynx salvage procedures [5–7], the National Comprehensive Cancer Network (NCCN) guidelines [8] etc.

For early laryngeal tumours, a single treatment modality is generally used, while for locally and locoregionally advanced tumours, a multimodal approach is recommended. Surgery is the standard treatment, with significant advances being made in endoscopic techniques in recent decades, and in many cases can replace external approaches [5]. Technological advances have led to improved treatment outcomes in this area, and surgical management preserving laryngeal function is now the preferred modality for early stages [4, 5, 9]. Nevertheless, the effect of radiotherapy in the treatment of early stages is supported by strong evidence and remains an important treatment of choice [10, 11].

In advanced stages, surgical procedures result in a significant mutilation of the patient when performing a total laryngectomy. Conditional to the extent of tumour involvement, the patient’s general condition and preferences, radiotherapy or chemoradiotherapy are the methods of choice as organ-preserving treatment modalities to provide comparable tumour control while allowing laryngeal preservation in 60–70% of patients [4]. The essential evaluated parameter is not simply the preservation of the larynx itself, but its functionality to ensure a better quality of life. The laryngo-esophageal dysfunction-free survival rate (LEDFS), *i.e.*, the proportion of patients not in need of securing the airway or swallowing, is evaluated [4, 5]. Thanks to the technological advances in diagnostic and radiotherapeutic methods in recent decades, better outcomes following radiation treatment can be achieved with lower acute and late side effects.

The aim of this report is to present the impact of comorbidities and lifestyle factors on the treatment outcomes in a cohort of patients irradiated for laryngeal cancer at the Institute of Radiation Oncology of Bulovka University Hospital (IRO BUH) in the years 2009–2018.

## Materials and methods

During the years 2009–2018, 189 patients aged 37–82 years (median age 63 years) underwent curative radiotherapy for laryngeal cancer at IRO BUH; 160 were male and 29 were female. Nicotinism and alcohol abuse were present in 172 (91.0%) and 155 (82.0%) patients, respectively. The characteristics of the patient cohort are shown in Table 1. Comorbidities were ascertained in patients at the standard initial examination and the Charlson Comorbidity Index (CCI) [12, 13], the Adult Comorbidity Evaluation 27 (ACE-27) score [14] and the American Society of Anesthesiologists (ASA) classification [15] were calculated for research purposes.

**Table 1:** Patients, tumour and treatment characteristics. Median (range) is reported for continuous and counts (percentage) for categorical variables

Variable	Group	Number of patients (%)/Median (range)
*Gender*	Female	29 (15.3%)
	Male	160 (84.7%)
*Mean age (years)*	–	64 (37–82)
*Histology grading*	G1/G2	127 (67.2%)
	G3/G4	61 (32.3%)
	0	1 (0.5%)
*Clinical stage* (Union for International Cancer Control/UICC/, 7th edition)	0is	1 (0.5%)
	I	47 (24.9%)
	II	40 (21.1%)
	III	44 (23.3%)
	IV	57 (30.2%)
*Primary tumour site*	Supraglottis	30 (15.9%)
	Glottis-supraglottis	8 (4.2%)
	Glottis	91 (48.2%)
	Glottis-subglottis	11 (5.8%)
	Subglottis	1 (0.5%)
	Transglottic	48 (25.4%)
*Neoadjuvant chemotherapy*	Yes	0
	No	100%
*Concurrent chemo/bio/radiotherapy*	Cisplatin tri-weekly	0
	Cisplatin weekly	47 (24.9%)
	Cetuximab	2 (1.0%)
	RT alone	140 (74.1%)
*PEG*	Yes	84 (44.4%)
	No	105 (55.6%)
*TRST*	Yes	90 (47.6%)
	No	99 (52.4%)
*Smoking history*	Yes	172 (91.0%)
	No	17 (9.0%)
*Alcohol abuse history*	Yes	151 (79.9%)
	No	38 (20.1%)
*Performance status*	0,1	161 (85.2%)
	2,3	28 (14.8%)
*Weight loss*	Weight loss < 1 kg	146 (77.2%)
	Weight loss < 10 kg	31 (16.4%)
	Weight loss ≥ 10 kg	12 (6.4%)
*Charlson Comorbidity Index (CCI)*	0–3	116 (61.4%)
	4–10	73 (38.6%)
*ACE-27 comorbidity score*	0,1	108 (57.1%)
	2,3	81 (42.9%)
*ASA score*	0, I, II	145 (76.7%)
	III, IV	44 (23.3%)
*Haemoglobin level (g/dl)*	Normal or elevated	120 (63.5%)
	Reduced	69 (36.5%)
*Second primary tumour*	Yes	23 (12.2%)
	No	166 (87.8%)
*Marital status*	Single	37 (19.6%)
	Married	101 (53.5%)
	Divorced	39 (20.6%)
	Widowed	12 (6.3%)
*Education*	Primary school	146 (77.2%)
	Middle school	33 (17.5%)
	University	10 (5.3%)
*Waiting time (days)*	–	64 (18–315)
*Radiotherapy treatment time (days)*	–	51 (37–72)

*T* stage Primary Tumour according to UICC 7th edition, *N* stage Regional Lymph Nodes according to UICC 7th edition, *G* tumour grading, *RT* radiotherapy, *ASA* American Society of Anesthesiologists, *ACE-27* Adult Comorbidity Evaluation 27, *PEG* percutaneous endoscopic gastrostomy tube, *TRST* tracheostomy

Patients received treatment in accordance with the standard guidelines in force at the time. In the early stages, irradiation was performed on the laryngeal region only with a dose of 70 Gy in 35 fractions in a normofractionated mode, whereas in the advanced stages, radiotherapy was performed in the area of laryngeal involvement and in the lymphatic areas with a dose of 56 Gy in 27 fractions, with a boost to high-risk areas for a total dose of 70 Gy in 35 fractions. Concomitant chemotherapy (cisplatin weekly), according to the current indication criteria (advanced disease, good overall condition without comorbidities preventing the addition of concomitant chemotherapy), was added for 48 (25.4%) patients. Patients designated for stand-alone radiotherapy were confirmed to have less advanced stages of disease (T1‑2 N0M0) or were unable to receive concomitant chemotherapy due to their worsened overall condition or comorbidities. A total of 179 (94.7%) patients completed their treatment.

Acute and late toxicity were evaluated according to RTOG (Radiation Therapy Oncology Group) criteria [16].

### Statistical analysis

As we were investigating the influence of comorbidities, lifestyle factors and other patient-related factors concerning survival, the endpoints were OS (Overall Survival), PFS (Progression Free Survival) and LC (Local Control). OS was defined as the interval commencing from the time of diagnosis to the final clinical follow-up or death. PFS was defined as the interval starting from the time of diagnosis to the local or distant disease progression, the last clinical follow-up or death. LC was defined as the time from the start of irradiation to the last clinical follow-up (in patients with remission) or to the date of local progression of the primary tumour or regional lymph nodes.

The data were analysed with SPSS statistical software, version 28, and *p*-values of less than 0.05 were considered significant. Univariate analyses of survival were carried out using the Kaplan-Meier method and the evaluation of differences between the groups was performed with the log-rank test. Univariate Cox proportional hazards regression analyses were performed to calculate HRs and CIs to evaluate the influence of the factors on risk of mortality or recurrence. A multivariate analysis of endpoints and prognostic factors was performed with the Cox proportional-hazards regression model and applying the forward stepwise method to define the independent contribution of each prognostic factor.

The Chi-square test and Student t‑test were used for categorical and continuous variables, respectively, to evaluate differences in risk factors between groups.

## Results

### Survival parameters

The median OS was 50.8 months, mean PFS was 96.5 months (median not reached), mean LC was 101.4 months (median not reached) and median follow-up time was 38.1 months (2.9–148.5 months). Persistence or recurrence was confirmed in 59 (31.2%) patients, a duplicate malignant tumour developed in 23 (12.2%) patients. Local recurrence/persistence of the disease was confirmed in 39 (20.6%) cases, 11 (5.8%) patients developed distant metastases with ongoing locoregional control of the disease and 9 (4.8%) patients developed synchronous local recurrence and distant metastases. LC was confirmed in 133 (70.4%) patients.

During the follow-up period, 120 (63.5%) patients died; the cause of death was disease progression in 47 (39.2%) patients, a duplicate malignant tumour in 15 (12.5%) patients, and other or unknown cause in 58 (48.3%) cases.

### Treatment toxicity

Acute treatment toxicity of grades 0–2, according to the RTOG criteria, was observed in 115 (60.8%) patients, while acute adverse effects of more severe intensity (grades 3–4) were observed in 74 (39.2%) patients with the following distribution: severe dysphagia in 26 (13.8%) cases, haematological toxicity in 23 (12.2%) patients, a skin reaction in 23 (12.2%) cases and mucositis in 12 (6.4%) cases.

Late toxicity was evaluated in a subset of 109 (57.7%) patients with a follow-up time of at least one year and sufficient documentation. Late toxicity of grade G3–4, according to the RTOG criteria, occurred in 11 (10.1%) patients: swallowing dysfunction in 4 (3.7%) cases, laryngeal dysfunction in 4 (3.7%) patients, xerostomia in 3 (2.8%) cases, skin or subcutaneous changes in 2 (1.8%) cases and osteonecrosis of the jaw in 1 (0.9%) patient.

A lower incidence of severe toxicity was observed in the subgroup of patients with early disease and smaller irradiated volume compared to the subgroup of patients with more advanced disease and larger irradiated volume. Grade 3–4 acute toxicity was observed in 15.9% of clinical stage (CS) I–II patients and 59.4% of CS III–IV cases. Late grade 3–4 toxicity developed in 5.5% of CS I–II patients and 14.8% of CS III–IV subgroup.

### Factors influencing survival

**Fig. 1:**
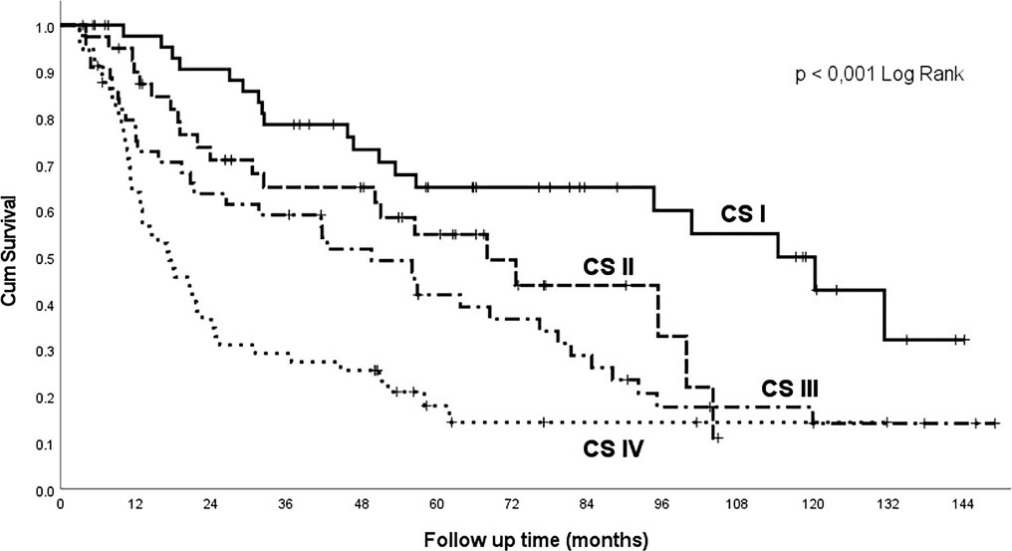
Overall survival by clinical stage. *CS* clinical stage according to UICC (Union for International Cancer Control), 7th edition

Overall survival by clinical stages, according to the Union for International Cancer Control (UICC), 7th edition, is shown in the Kaplan-Meier curves in Fig. 1. A statistically significant higher prevalence of more advanced clinical stages (clinical stages III–IV) was confirmed in patients in worse overall condition with PS ECOG 2–3 (performance status set according to the Eastern Cooperative Oncology Group): 86.2% compared to 47.3% in patients with initial PS 0–1 (*p* = 0.002).

**Fig. 2:**
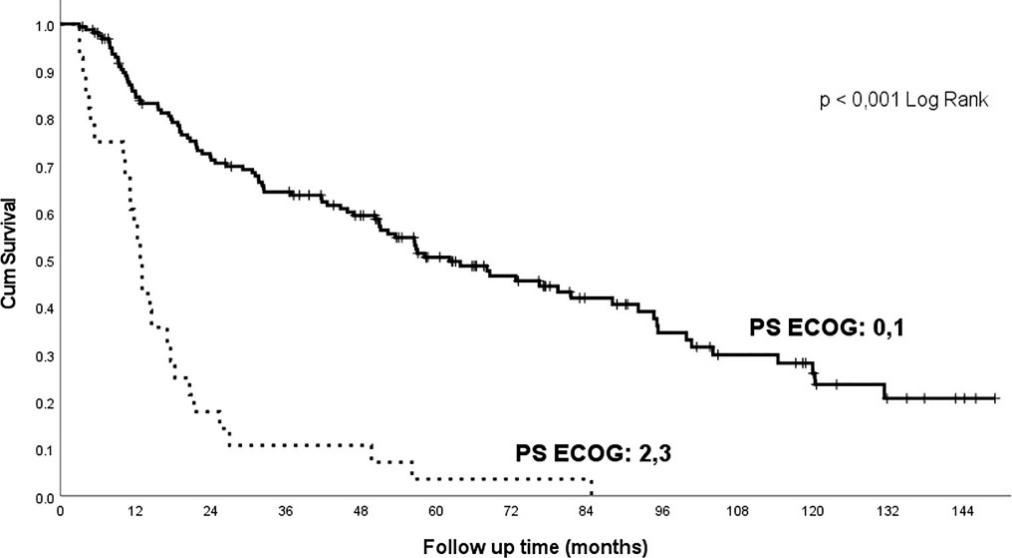
Overall survival related to the patient’s initial performance status (PS ECOG). *PS* performance status, *ECOG* Eastern Cooperative Oncology Group

**Fig. 3:**
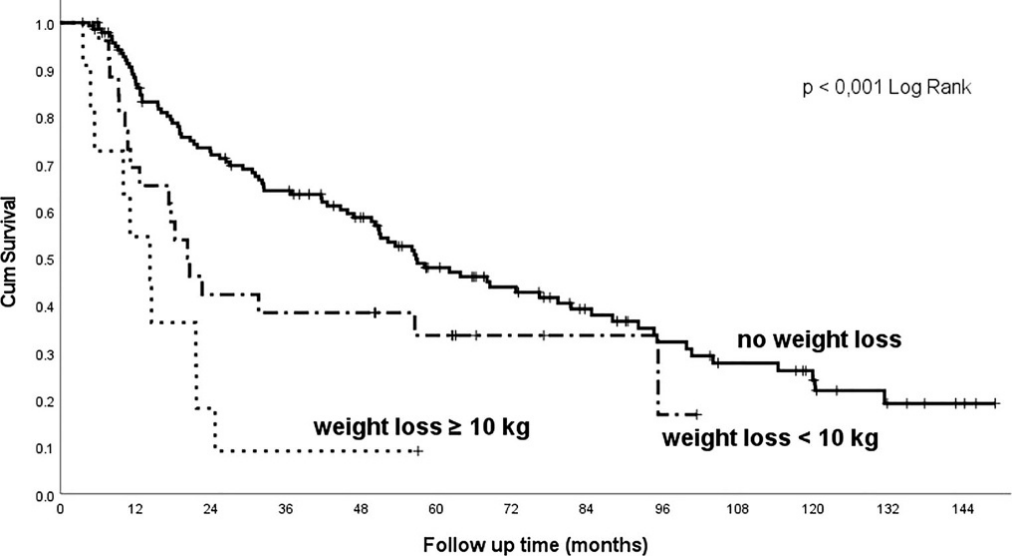
Overall survival related to pretreatment weight loss

In addition to the influence of commonly evaluated factors (clinical stage, histopathological grading, concomitant chemotherapy and tracheostomy insertion before treatment), our cohort demonstrated a highly significant influence of comorbidities and lifestyle factors on treatment outcomes. Significant influences are the baseline PS ECOG (Fig. 2), weight loss (Fig. 3) and haemoglobin values: patients in good condition, without major weight loss or without anaemia at baseline, survived significantly longer than patients in poor condition, with anaemia, or in patients with marked weight loss (Table 2).

**Table 2:** Univariate Cox proportional hazards regressions analyses for overall survival (OS), loco-regional control (LC), disease-free survival (DFS)

Prognostic factors	OS univariate analysis	PFS univariate analysis	LC univariate analysis
	HR (95% CI), *p* value	HR (95% CI), *p* value	HR (95% CI), *p* value
Gender (F vs M)	0.627 (0.357–1.100), *p* = 0.104	1.022 (0.717–1.457), *p* = 0.905	1.025 (0.687–1.531), *p* = 0.903
Age (years)	**1.022 (1.003–1.041), *p*** **=** **0.024**	0.986 (0.960–1.012), *p* = 0.278	0.974 (0.946–1.003), *p* = 0.079
Overall radiotherapy treatment time TT (days)	0.978 (0.946–1.011), *p* = 0.185	1.007 (0.963–1.052), *p* = 0.768	1.005 (0.958–1.055), *p* = 0.834
Clinical stage (UICC 7th edition)	**1.635 (1.379–1.937), *p*** **<** **0.001**	**1.568 (1.233–1.994), *p*** **<** **0.001**	**1.497 (1.150–1.948), *p*** **=** **0.003**
Histology grading G1/G2 vs G3/G4	**1.034 (1.011–1.057), *p*** **=** **0.003**	1.024 (0.993–1.056), *p* = 0.133	1.015 (0.980–1.051), *p* = 0.407
ASA	**1.771 (1.287–2.438), *p*** **<** **0.001**	1.192 (0.717‑1.982), *p* = 0.499	1.376 (0.789–2.399), *p* = 0.260
Charlson comorbidity index (CCI)	**1.149 (1.064–1.241), *p*** **<** **0.001**	0.989 (0.872–1.122), *p* = 0.867	0.980 (0.851–1.127), *p* = 0.775
ACE-27	**1.439 (1.177–1.759), *p*** **<** **0.001**	1.185 (0.884–1.589), *p* = 0.255	1.286 (0.931–1.776), *p* = 0.128
PS ECOG (0 a 1 vs 2 a 3)	**2.761 (1.897–4.019), *p*** **<** **0.001**	**1.104 (1.054–1.157), *p*** **<** **0.001**	**1.090 (1.032–1.150), *p*** **=** **0.002**
Haemoglobin concentration (g/dl) (anaemia vs normal level)	**0.975 (0.965–0.986), *p*** **<** **0.001**	0.640 (0.372–1.100), *p* = 0.106	0.846 (0.450–1.592), *p* = 0.604
RT dose to the tumour (Gy)	**0.941 (0.920–0.963), *p*** **<** **0.001**	0.964 (0.922–1.007), *p* = 0.102	0.961 (0.921–1.002), *p* = 0.063
PEG no/yes	**1.279 (1.068–1.530), *p*** **=** **0.007**	**1.340 (1.036–1.733), *p*** **=** **0.026**	**1.345 (1.011–1.790), *p*** **=** **0.042**
TRST no/yes	**2.513 (1.737–3.635), *p*** **<** **0.001**	**2.239 (1.324–3.784), *p*** **=** **0.003**	**2.193 (1.227–3.917), *p*** **=** **0.008**
Larynx subsite tumour location (glottis, subglottis, supraglottic)	**1.007 (1.003–1.010), *p*** **<** **0.001**	**1.385 (1.134–1.692), *p*** **=** **0.001**	**1.391 (1.113–1.737), *p*** **=** **0.004**
Concurrent chemo/bio/radiotherapy:			
*RT alone vs Cisplatin weekly*	1.197 (0.803–1.783), *p* = 0.377	1.126 (0.701–2.143), *p* = 0.475	0.745 (0.352–1.578), *p* = 0.442
*RT alone vs Cetuximab*	2.404 (0.589–9.811), *p* = 0.221	**5.776 (1.387–24.049), *p*** **=** **0.016**	2.014 (0.535–7.582), *p* = 0.300
Smoking history (no/yes)	1.128 (0.589–2.157), *p* = 0.717	1.316 (0.477–3.635), *p* = 0.596	1.490 (0.463–4.801), *p* = 0.504
Alcohol abuse history (no/yes)	1.611 (0.945–2.747), *p* = 0.080	0.695 (0.381–1.269), *p* = 0.236	0.914 (0.442–1.891), *p* = 0.809
Weight loss before RT:			
< *1* *kg vs 1–10* *kg*	1.668 (0.999–2.785), *p* = 0.050	1.881 (0.987–3.586), *p* = 0.550	**2.323 (1.168–4.621), *p*** **=** **0.016**
*<* *1* *kg vs >* *10* *kg*	**4.174 (2.130–8.181),** ***p*** **<** **0.001**	**3.332 (1.406–7.899), *p*** **=** **0.006**	**3.820 (1.478–9.872), *p*** **=** **0.006**
Weight loss during RT:			
< *1* *kg vs 1–10* *kg*	1.077 (0.735–1.577), *p* = 0.704	0.830 (0.483–1.426), *p* = 0.500	0.884 (0.482–1.620), *p* = 0.690
*<* *1* *kg vs >* *10* *kg*	0.859 (0.422–1.752), *p* = 0.677	1.207 (0.501–2.909), *p* = 0.675	1.554 (0.630–3.835), *p* = 0.339
Second primary tumour	0.898 (0.536–1.503), *p* = 0.682	**0.281 (0.088–0.897), *p*** **=** **0.032**	0.343 (0.107–1.106), *p* = 0.073
Marital status:			
*Married vs divorced*	1.220 (0.762–1.954), *p* = 0.407	1.239 (0.639–2.401), *p* = 0.526	1.291 (0.626–2.633), *p* = 0.489
*Married vs widowed*	1.813 (0.896–3.667), *p* = 0.098	1.597 (0.557–4.578), *p* = 0.384	1.430 (0.427–4.792), *p* = 0.562
*Married vs single (never married*)	**1.938 (1.232–3.049), *p*** **=** **0.004**	**2.221 (1.177–4.190), *p*** **=** **0.014**	**2.194 (1.081–4.450), *p*** **=** **0.029**
Education:			
*Primary school vs middle school*	1.161 (0.743–1.814), *p* = 0.512	**1.827 (1.013–3.296), *p*** **=** **0.045**	1.766 (0.914–3.412), *p* = 0.090
*Primary school vs university*	1.151 (0.532–2.491), *p* = 0.721	0.765 (0.185–3.160), *p* = 0.711	0.884 (0.212–3.680), *p* = 0.866

Gender-*F*-females, *M*-men, *RT* radiotherapy, *Gy* (Gray), *HR* hazard ratio, *CI* confidence interval, *UICC* The Union for International Cancer Control, *G* grade, *PS* performance status, *ECOG* Eastern Cooperative Oncology Group, *ASA* American Society of Anesthesiologists, *ACE-27* Adult Comorbidity Evaluation 27, *PEG* percutaneous endoscopic gastrostomy tube, *TRST* tracheostomy

**Fig. 4:**
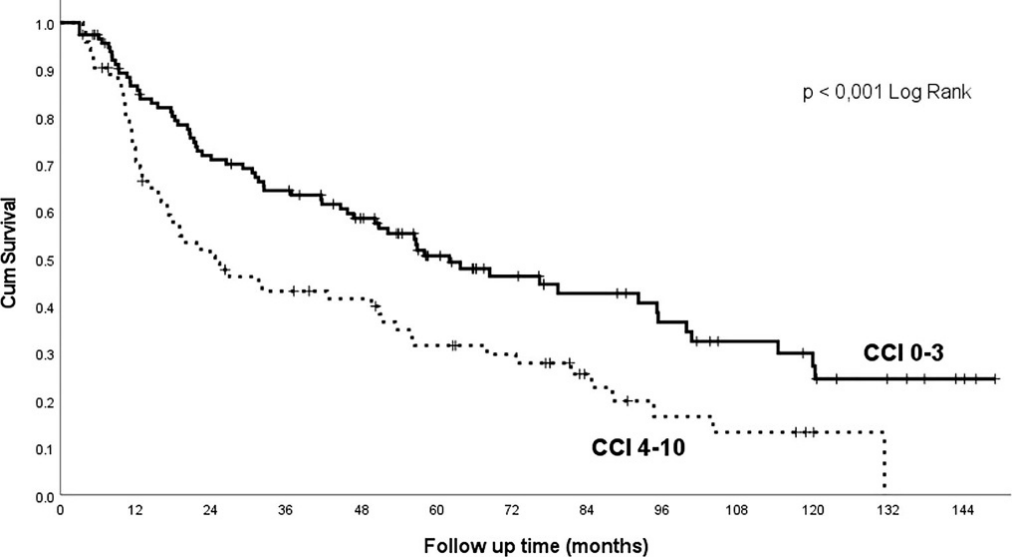
Overall survival related to the Charlson Comorbidity Index (CCI)

**Fig. 5:**
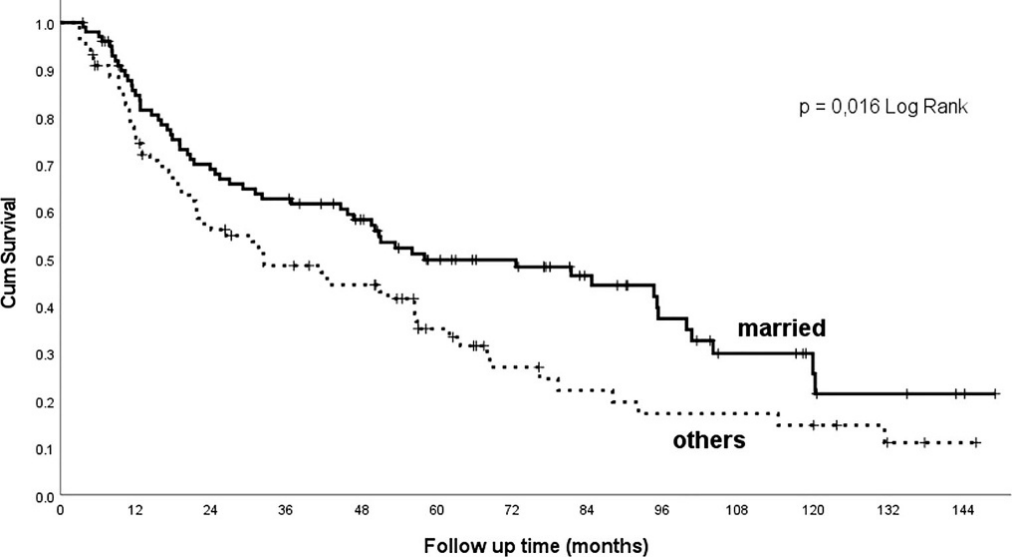
Overall survival by marital status

Comorbidity scores, according to the CCI (Fig. 4), the ACE-27 score and ASA classification, are of prognostic importance (Table 2). Alcohol abuse had a significant effect on outcomes (Table 2), whereas nicotinism had no significant effect in our cohort, probably due to a history of smoking in the vast majority (91%) of patients and the small size of the cohort. Marital status also has an impact on treatment outcomes in our cohort; married patients achieved significantly longer overall survival than non-married ones (Fig. 5, Table 2).

In the multivariate analysis, OS, PFS and LC were significantly affected by the clinical stage and PS (Table 3).

**Table 3:** Multivariate analyses for overall survival (OS), loco-regional control (LC), disease-free survival (DFS), only factors significant in univariate analysis were calculated

Prognostic factors	OS	PFS	LC
	HR (95% CI), *p* value	HR (95% CI), *p* value	HR (95% CI), *p* value
Gender (F vs M)	ND	ND	ND
Age (years)	NS	ND	ND
Overall radiotherapy treatment time TT (days)	**ND**	**ND**	**ND**
Clinical stage (UICC 8th edition)	**1.738 (1.384–2.182), *p*** **<** **0.001**	**1.500 (1.169–1.924), *p*** **=** **0.001**	**1.426 (1.088–1.869), *p*** **=** **0.010**
Histology grading G1/G2 vs G3/G4	NS	ND	ND
ASA	NS	ND	ND
Charlson comorbidity index (CCI)	1.157 (1.037–1.291), *p* = 0.009	ND	ND
ACE-27	NS	ND	ND
PS ECOG (01 vs 23)	**1.073 (1.025–1.123), *p*** **=** **0.002**	**2.851 (1.528–5.319), *p*** **<** **0.001**	**1.072 (1.014–1.133), *p*** **=** **0.015**
Hemoglobin concentration (g/dl) (anemia vs normal level)	NS	ND	ND
RT treatment dose to the tumour (Gy)	NS	ND	ND
PEG yes/no	NS	NS	NS
TRST yes/no	NS	NS	NS
Larynx subsite tumour location (glottis, subglottis, supraglottic)	NS	NS	NS
Concurrent chemo/bio/radiotherapy	ND	NS	ND
Smoking history (no/yes)	ND	ND	ND
Alcohol abuse history (no/yes)	ND	ND	ND
Weight loss before RT	NS	NS	NS
Weight loss during RT	ND	ND	ND
Second primary tumour	ND	NS	ND
Marital status	**1.241 (1.043–1.475), *p*** **=** **0.015**	NS	NS
Education:			
*Primary school vs middle school* *Primary school vs university*	ND	**1.992 (1.102–3.600), *p*** **=** **0.023** 0.688 (0.166–2.852), *p* = 0.607	ND

Gender-*F*-females, *M*-men, *RT* radiotherapy, *NS* not significant, *ND* not done, *HR* hazard ratio, *CI* confidence interval, *UICC* The Union for International Cancer Control, *G* grade, *PS* performance status, *ECOG* Eastern Cooperative Oncology Group, *ASA* American Society of Anesthesiologists, *ACE-27* Adult Comorbidity Evaluation 27, *PEG* percutaneous endoscopic gastrostomy tube, *TRST* tracheostomy

## Discussion

Curative radio(chemo)therapy of laryngeal tumours is associated with a relatively high rate of toxicity during treatment and afterwards. A significant proportion of patients develop late toxicity in the form of laryngeal dysfunction or severe swallowing disorders causing full or partial dependence on nutritional gastrostomy. Precise patient education, good patient cooperation and complete care during the follow-up are essential when managing complications to ensure a good quality of life. This treatment should, therefore, be performed in specialised centres with good facilities of otorhinolaryngological surgery, clinical oncology, radiation therapy and related disciplines (including the support of a gastroenterologist, nutritionist, clinical pharmacist, dermatologist, pneumologist, and psychosocial assistance).

IRO BUH has a long tradition of treating patients with head and neck cancer. The treatment approach is initially determined by a multidisciplinary team involving a clinical and radiation oncologist, an otorhinolaryngologist, a radiologist and a pathologist. Treatment options are discussed with the patient and the patient’s preferences are taken into consideration as treatment decisions are being made. Post-treatment follow-ups are also carried out interdisciplinarily at IRO BUH with the participation of a clinical and radiation oncologist and an otorhinolaryngologist.

During the evaluation period, 2009–2018, patients were irradiated with 3D-CRT (3D conformal radiotherapy) or IMRT (Intensity Modulated Radiation Therapy) in two phases with a normofractionation mode. With the implementation of new technologies in radiation oncology, it is possible to target the radiation better, adjust the dose in each area of the target volume and save surrounding healthy tissue. Currently, our patients with head and neck tumours are irradiated using the VMAT-SIB (Volumetric Modulated Arc Therapy) technique with the possibility of daily control of the irradiation position using CBCT (Cone Beam Computed Tomography)—the IGRT (Image Guided Radiation Therapy) technique. Lower rates of late treatment toxicity are expected in the future as a result of using these new technologies and techniques, a fact already reflected in a more satisfactory profile of the acute side effects of treatment.

Associated comorbidities at the time of the laryngeal cancer diagnosis, together with a common history of nicotinism and alcohol abuse, represent a significant burden in the form of higher risk of complications. Comorbidities have been shown to play a significant role in many aspects of care in elderly patients suffering from cancer, including the choice of treatment procedures which affect the response to therapy, tumour progression, morbidity, and survival outcomes [17]. Pre-treatment comorbidity status has been repeatedly confirmed as an independent predictive factor for overall survival [17–20]. The CCI demonstrated a strong role in predicting overall survival and non-cancer cause-specific survival in an analysis of data from 548 patients with laryngeal cancer but not associated with disease specific survival [17].

In that cohort, the authors found a 5-year survival rate of 60% in patients with CCI > 3 and 41% in patients with CCI 0–3; in our cohort, 5‑year survival rates of 54% and 32% were achieved in comparable subgroups (Fig. 4).

In a cohort of 180 patients with laryngeal cancer [19], including patients referred to surgery, radiotherapy, or combined therapy, the overall 5‑year survival rate was 65%. Based on the clinical stage (CS), the 5‑year survival rate was: CS I 69%, CS II 81%, CS III 53%, CS IV 25%. The 5‑year survival rate according to the ACE-27 comorbidity score was: no comorbidities 82%, mild 61%, moderate 37%, and severe comorbidities 18%. In our cohort, the following values were achieved: overall 5‑year survival rate 43%, 5‑year survival rate according to clinical stages: stage I 65%, II 56%, III 41%, IVA 21%, IVB 0% (Fig. 1). The 5‑year survival rate according to ACE-27: stage 0–1 (no comorbidities or mild comorbidities) 50%, stage 2–3 (moderate or severe comorbidities) 33%.

Overall, the 2‑year survival rate in our cohort was 62%, and the Veterans Affairs Laryngeal Cancer Study Group [21] reported an estimated 2‑year survival rate of 68%. The differences between our results and the literature data may be due to a different spectrum of patients, including a higher prevalence of comorbidities, a worse initial PS ECOG of the patients and other adverse factors. The higher rate of comorbidities compared to the literature data may be caused by regional specificities of the population or the extent of the examination of patients prior to irradiation. The poorer general condition of patients is also associated with a lower frequency of concomitant chemotherapy in our cohort—74% of patients underwent radiotherapy alone without chemotherapy.

A strong prognostic impact of comorbidities has been demonstrated in multiple analyses of cohorts of patients with laryngeal cancer [11, 17, 19], head and neck cancers, in general [18], and a variety of other cancers [21]. Consistent with the literature, the results of our cohort of patients confirmed the prognostic impact of comorbidities assessed by the CCI (in particular, the significant role of patient age), the ACE-27 score (ability to score according to the severity of associated diseases) and the ASA system (scores for anaesthesiologists, a different way of assessing comorbidities and nicotinism, and lower stratification of comorbidity severity compared to the CCI and ACE-27).

The importance of haemoglobin levels before and during radio(chemo)therapy for predicting survival has been investigated [11, 22, 23]. A low haemoglobin level before treatment had a negative impact on local control and disease-specific survival in a set of elderly patients with early glottic cancer [11]. The evolution of haemoglobin values during radio(chemo)therapy [22], or at the end of cancer treatment [23], seems to be more important than baseline values.

In our cohort of patients with laryngeal cancer, the effect of baseline PS, marked weight loss and haemoglobin level on overall survival was confirmed to be as significant as the effect of the clinical stage. Changes in haemoglobin levels during radio(chemo)therapy and its influence on overall survival were not analysed.

The gross tumour volume (GTV) and the radiotherapy treatment time confirmed a significant prognostic effect in patient cohorts [24, 25], as well as the waiting time between collecting tumour specimen and the beginning of radiotherapy [11]. However, the waiting time or overall treatment time did not affect the outcomes in our cohort and GTV size was not analysed.

These findings support the individual consideration of treatment, as tailored treatments, based on many parameters, can bring maximum benefits to the patient.

## Conclusions

In our cohort of patients treated with radio(chemo)therapy for laryngeal cancer, we found good therapeutic results and an acceptable side-effect profile. Our findings confirmed the statistically significant effect of comorbidities, history of alcohol abuse and marital status on a patient’s overall survival rate. The prognostic potential of baseline PS, the weight loss and haemoglobin levels were also validated. Due to the complexity of radical radio(chemo)therapy for laryngeal cancer associated with significant acute and late toxicity, tailored treatments, based on evidence-based medicine with multidisciplinary supportive care during treatment and follow-up, is essential to achieve maximum therapeutic benefits and ensure a good quality of life for patients.
